# Negative Problems Orientation Questionnaire for Chinese Adolescents: Bifactor Model and Measurement Invariance

**DOI:** 10.3389/fpsyg.2020.608676

**Published:** 2020-12-10

**Authors:** Huiwen Xiao, Rongmao Lin, Qiaoling Wu, Saili Shen, Youwei Yan

**Affiliations:** ^1^School of Psychology, Fujian Normal University, Fuzhou, China; ^2^Guilin University of Aerospace Technology, Guilin, China

**Keywords:** Negative Problem Orientation Questionnaire, negative problem orientation, bifactor model, measurement invariance, adolescents, China

## Abstract

The Negative Problem Orientation Questionnaire (NPOQ) is a widely used tool for assessing negative problem orientation (NPO). However, its construct and measurement invariance has not been adequately tested in adolescents. The present study explored the possible construct of the NPOQ and its measurement invariance in a sample of 754 Chinese adolescents (51.6% girls, all 12–18 years old). The results supported a bifactor model of the NPOQ that consists of a general factor NPO and three domain-specific factors including perceived threat, self-inefficacy, and negative outcome expectancy. A multiple-group CFA indicated that the bifactor model showed strict invariance across gender and age. The general and domain factors showed unique variance in indexes of worry, depression, anxiety, and stress, which supported well incremental validity of them. This study confirms for a bifactor conceptualization of the NPOQ and its measurement invariance across gender and age in Chinese adolescents. Additionally, it is recommended that the total score should be used to assess NPO in Chinese adolescents.

## Introduction

Negative problem orientation (NPO) is an important construct in our understanding of deficits in problem-solving ability as manifested in daily life. It refers to a set dysfunctional attitudes regarding social problem solving, which increases worry about social problems and inhibits effective resolution (Nezu and D’Zurilla, 2006; Bell and D’Zurilla, 2009). Indeed, NPO not only results in poor social problem solving, but also uniquely links to many mental health disorders, for example, generalized anxiety disorder (Dupuy and Ladouceur, 2008), social anxiety disorder (Hearn et al., 2017; Romano et al., 2019), depressive disorder (Becker-Weidman et al., 2010; Anderson et al., 2011; Balsamo et al., 2013), and eating disorders (Konstantellou et al., 2011).

Considered its important role in social and health problems, how to measure NPO is vital in health and clinical context. Since its publication in 2005, the Negative Problem Orientation Questionnaire (NPOQ) has emerged as the most widely used self-report measure of NPO. It uses 12 items to assess negative beliefs regarding social problems on a 5-point scale (ranging from 1 to 5). It shows excellent internal consistency (α = 0.92), good test-retest reliability (*r* = 0.80, over interval 5 weeks), and demonstrates both convergent and discriminant validity (Robichaud and Dugas, 2005a,b). Over the past 15 years, the NPOQ has been used in most psychosocial studies of NPO including clinical evaluation and diagnosis of anxiety and mood disorders (Dupuy and Ladouceur, 2008; Fergus and Wu, 2010), treatment outcome trails (Humphrey, 2016; Erdley-Kass et al., 2018), and also assessment of the social problem solving-ability in adolescent population (Barahmand, 2008; Zumberg et al., 2008). Despite the popularity and empirical utility of the NPOQ, its construct validity and measurement invariance across age and gender have not been sufficiently tested in adolescents.

### Construct Validity: From a Bifactor Perspective

The construct validity of NPOQ is relatively few supported. Robichaud and Dugas (2005a,b), which relied on two samples of university students (*N* = 201, 148), found only one factor in NPOQ by principal components factor analysis and maximum likelihood factor analysis, accounting for 54.8 and 52% of the variance, respectively. So far, the unitary factor model only tested in adult sample, but has yet been validated in adolescents. Previous studies have showed that adolescents experience an increasingly NPO trajectory, differing from the relatively stable level in adults (Ciarrochi et al., 2009). Furthermore, the one factor structure may not fully reflect the concept of NPO. As the social problem-solving theory described, NPO is a general negative disposition toward problems that can lead to negative emotions, avoidance, increased worry, and a reduction in effort to resolve complications (D’Zurilla et al., 2004). Specifically, it refers to a disruptive cognitive-emotional set or attitude toward social problems that involves three components: perceived threats to wellbeing, self-inefficacy or doubt regarding one’s problem-solving ability, and a tendency to be pessimistic about outcomes (D’Zurilla et al., 1998; Hawkins et al., 2009). From the social problem-solving model, a three-factor construct may be better described the NPOQ.

Whether NPOQ shows as a unitary or three-factor construct in adolescents? To answer the question, this study firstly compares the unitary and three-factor models via traditional correlated-factor models in confirmatory factor analysis, and further determines from a bifactor perspective. The bifactor model, which is also named as general-specific factor model, describes that there is a general factor representing the common variance of all the items and there are some group factors that account for additional common variance of all items after controlling the general factor (Rodriguez et al., 2016a,b). In the bifactor model, the general factor represents the conceptually broad “target” construct an instrument was designed to measure, and the group factors represent more conceptually narrow subdomain constructs (Reise, 2012). In contrast to the traditional correlated-factors model that must compare the unitary and multidimensional constructs in different models, the bifactor model can simultaneously estimate the different role of general factor and group factors in a bifactor model, it further determines the assumption that the group factors capture unique variances after accounting for the common variances or alternatively that the general factor mainly captures the common variance (Reise, 2012; Reise et al., 2016; Rodriguez et al., 2016b). The factor loadings on the general factor and group factors also can be considered to evaluate the validity of the bifactor model (Nadort et al., 2009; Saggino et al., 2018). In the study, performing a bifactor model is helpful to validate whether three group factors of NPOQ capture unique variances after accounting for the common variances or that the NPOQ manifests in a single construct.

### Measurement Invariance Across Age and Gender

Another potential weakness in common practices of using NPOQ is the lack of accounting adequately for the measurement error. Prior studies indicated that adolescent NPO may differ profoundly by gender and age (Barahmand, 2008; Ciarrochi et al., 2009). For example, Barahmand (2008) found that boys reported greater NPO than girls. Also, the findings revealed certain age trends that the NPO may be under the influence of developmental tasks (Barahmand, 2008). Similarly, with a latent growth analysis, Ciarrochi et al. (2009) showed that adolescents experienced an increasingly NPO trajectory as their age. However, these researches have not considered the implications of measurement invariance when making group comparisons.

Measurement invariance generally refers to the extent to which the content of each item is being perceived and interpreted in the same way across samples (Vandenberg and Lance, 2000). In fact, if measures of NPO operate differently across gender and age groups and these variations are not taken into account in the measurement, it is inappropriate to compare levels of NPO across groups (Cheung, 2008; Schmitt and Kuljanin, 2008). Thus, measurement invariance is precondition for group comparisons. Testing for measurement invariance includes a series of hierarchical steps, beginning with the establishment of a baseline model in each group, followed by tests for equivalence across groups at several increasingly more restricted levels including but not limited at configural, factor loadings, indicator thresholds and indicator residual errors (Vandenberg and Lance, 2000; Schmitt and Kuljanin, 2008; Carlucci et al., 2018).

### The Current Study

In sum, though NPOQ has been widely applied to assessment of adolescent’s NPO, its construct validity and measurement invariance have not been sufficiently supported. The main purpose of the present work is to explore the factor structure of the NPOQ and test its measurement invariance with Chinese adolescents. In addition, this research also tests the incremental validity of the NPOQ for Chinese adolescents. An increasing number of studies have shown that NPO is an important predictive factor for worry and anxiety (Kertz and Woodruff-Borden, 2012; for a review, see Clarke et al., 2017). In the cognitive model of anxiety disorder, Dugas et al. (2005) proposed that NPO is the key cognitive factor in chronic anxiety and worry. Also, patients with depressive symptoms tend to use more NPO (Bell and D’Zurilla, 2009). Thus, the present study uses worry, anxiety, depression, and related stress as criteria.

## Materials and Methods

### Participants

A sample of 855 adolescents was recruited via a stratified cluster sampling, which each class was saw as a cluster, and the grade was saw as level (included 4 grades). In this survey, four classes were sampled in each grade, and a total of 16 classes were recruited from four public middle schools in Fuzhou, Fujian Province. The total number of participants for each school were 188 (25%), 171 (22.7%), 203 (27%), and 192 (25.5%), respectively. All participants completed the questionnaire in class, which was administrated by their head teacher and a postgraduate majoring in psychology. Approval was obtained from participants’ head teachers and parents. A primary check was conducted when the responses were collected; participants who responded to the same option for more than half of the questionnaire or blank were excluded. Finally, an available sample of 754 adolescents was retained (the rate of availability was 88.2%). Participants ranged in age from 12 to 18 years (*M* = 14.9, *SD* = 1.6). In the available sample, 365 were boys (48.4%) and 389 were girls (51.6%). The numbers of students in the 7th, 8th, 10th, and 11th grades were 188 (25%), 171 (22.7%), 203 (27%), and 192 (25.5%), respectively. This study was approved by the Academic Committee of Fujian Normal University.

### Measures

#### Negative Problem Orientation Questionnaire (NPOQ)

NPOQ is a 12-item measure that assesses a dysfunctional cognitive set that includes the tendency to see problems as a threat, to doubt one’s own problem-solving ability, and to be pessimistic about the outcome (Robichaud and Dugas, 2005a,b). It was shown to have excellent internal consistency (α = 0.92), good test-retest reliability (*r* = 0.80, interval 5 weeks), and demonstrated convergent (*r* = 0.83, *p* < 0.001) and discriminant validity (*r* = 0.40–0.42, *p*_*s*_< 0.001). After obtaining permission from the authors, the original version of the NPOQ was translated into Chinese. The work was conducted via four independent bilingual translators, two whose native language was Chinese and who majored in psychology translated the English version into Chinese, and two bilingual translators whose native language was English translated the Chinese back into English. The translation process was repeated until discrepancies between the original and reverse-translated versions were negligible. Finally, the Chinese version of the NPOQ was proposed and used to test the psychometric properties of it in adolescents.

#### Depression-Anxiety-Stress Scale

The Depression-Anxiety-Stress Scale (DASS-21) includes three subscales: depression, anxiety and stress. Each subscale incorporates seven items. Participants respond to each by rating the frequency and severity of symptoms experienced in the previous week on a 4-point Likert scale ranging from 0 (“did not apply to me at all”) to 3 (“applied to me very much or most of the time”). Higher scores are indicative of higher levels of negative emotionality. In the current study, the Cronbach’s alpha coefficient of the DASS-21 was 0.94; those of the depression, anxiety, and stress subscales were 0.88, 0.85, and 0.85, respectively.

#### Penn State Worry Questionnaire

PSWQ is a 16-item questionnaire developed by Meyer et al. (1990). PSWQ measures both frequency and intensity of worry, with participant responding on 5-point Likert scale (from 1 to 5, indicates “not typical at all,” “very typical,” respectively). The total score ranges from 16 to 80, and higher scores indicate a greater tendency to feel worry. In the current study, the Cronbach’s alpha coefficient for the PSWQ was 0.84, and the coefficients of the subscales of “engaging in worry” and “absence of worry” were 0.92 and 0.70, respectively.

#### Data Analytic Strategy

##### Measurement Model

Three models were compared by CFA: unitary factor, correlated three-factor, and bifactor. In the unitary factor model, all items were loaded onto one factor. The second model was a correlated three-factor model consisting of three items (1, 8, and 12) with primary loading on Factor 1 (perceived threat), four items (2, 3, 5, and 9) with primary loading on Factor 2 (self-inefficacy), and five items (4, 6, 7, 10, and 11) with primary loading on Factor 3 (negative outcome expectancy). The third model was bifactor model: all 12 items were freely loaded onto a general factor and respective domain-specific factors. The dimensions of the group factor were the same as the classifications for the three-factor model. The covariance of all factors was fixed to zero in the bifactor model (Rodriguez et al., 2016b).

##### Measurement Invariance

Measurement invariance testing was accomplished via a multiple-group CFA. The best fitting model was tested for different genders and age to ensure that the same measurement model was supported for each group. Next, restrictive models were used to test for: (a) configural invariance (equal forms), (b) metric invariance (equal factor loadings), (c) scalar invariance (equal indicator thresholds), and (d) error invariance (equal indicator residual errors).

##### Structural Regression Model

A structural regression model was used to test if the factors of the NPOQ were related to the PSWQ and DASS. The structural regression model consisted of simultaneously modeling the selected model for the NPOQ described above, a bifactor model of the PSWQ (Zhong et al., 2009), and a three-factor model of the DASS (Henry and Crawford, 2005). Pathway coefficients were freely estimated from factors of the NPOQ for the PSWQ and DASS.

##### Model Estimation and Evaluation

All models were tested with Mplus version 7.4 for windows, and were estimated by a maximum likelihood estimation procedure (ML) and principal axis factoring (PAF). When evaluated individual model fit, the chi-square test (*χ^2^*), the normed chi-square test (*χ^2^*/*df*), and multiple complementary fit indices were considered. The chi-square test and normed chi-square test are significantly influenced by sample sizes (Hu and Bentler, 1998; Wen et al., 2004). However, results less than 5 for *χ^2^*/*df* usually indicate acceptability (Wen et al., 2004). Multiple complementary fit indices including root mean square error of approximation (*RMSEA*), Tucker-Lewis Index (*TLI*), Comparative Fit Index (*CFI*), and Standardized Root Mean Square Residual (*SRMR*) were used to evaluate model fit (Hu and Bentler, 1999). The following criteria were used to evaluate fit: *CFI* and *TLI* should be equal to or larger than 0.90, RMSEA and SRMR should be equal to or smaller than 0.08 (Hu and Bentler, 1999). When compared the nested models in multiple-group CFA, The indicators mentioned above will also be used. And the difference in *CFI* is also recommended. A value of △*CFI* smaller than or equal to 0.01 indicates rationally trivial differences in parameter estimates among models (Cheung and Rensvold, 2002; Meade et al., 2008).

## Results

### NPOQ Measurement Models

Data-screening analysis was conducted to detect items with skewed and kurtosis distribution. As shown in Table 1, the means of each item ranged from 1.98 to 2.86, with standard deviations from 1.06 to 1.42. The amount of skewness ranged from 0.00 to 0.97, and Kurtosis was from −1.37 to 0.184, suggesting that there was no outlier with extreme non-moral distribution.

**TABLE 1:** Descriptive statistics of negative problem orientation questionnaire in Chinese adolescents (*N* = 754).

Items	*M* ± *SD*	Skewness	Kurtosis
Item 1	2.04 ± 1.20	0.804	−0.577
Item 2	2.60 ± 1.33	0.217	−1.258
Item 3	2.29 ± 1.21	0.656	−0.589
Item 4	2.00 ± 1.06	0.939	0.184
Item 5	2.41 ± 1.28	0.477	−0.964
Item 6	2.09 ± 1.19	0.940	−0.090
Item 7	2.26 ± 1.34	0.690	−0.874
Item 8	2.05 ± 1.23	0.925	−0.265
Item 9	2.13 ± 1.28	0.820	−0.579
Item 10	2.86 ± 1.42	0.002	−1.373
Item 11	2.58 ± 1.32	0.301	−1.170
Item 12	1.98 ± 1.17	0.968	−0.075

Goodness-of-fit statistics for the measurement models appear in Table 2. Both the unitary factor model (Model 1) and correlated three-factor model (Model 2) did not achieve the specified guidelines. For the bifactor model (Model 3), *χ^2^* was significant, but *χ^2^*/*df* was less than 5 (Wen et al., 2004); CFI, TLI, RMSEA, and SRMR achieved the specified guidelines. The bifactor model produced the most favorable fit statistics than others. The factor loadings are presented in Table 3. Almost 12 items on the NPOQ continued to robustly load onto their respective domain-specific factors after controlling for the general factor. The loading on the general factor was nearly larger than the loadings on the domain-specific factors. Moreover, the factor loadings for items 4 and 6 for Factor 3 (negative outcome expectancy) were negative, while the remaining items loaded positively.

**TABLE 2:** Goodness-of-fit statistics for tested models.

Model	*χ ^2^*	*df*	*χ ^2^*/*df*	*CFI*	*TLI*	*RMSEA*	*SRMR*	Model comparison	Δ*χ ^2^(Δ df)*	Δ*χ ^2^*/Δ*df*	Δ*CFI*
**Single group (*n* = 754)**											
Model 1: one-factor	599.02	54	11.093	0.876	0.849	0.116	0.057	–	–	–	–
Model 2: three-factor	350.591	51	6.874	0.932	0.912	0.088	0.042	–	–	–	–
Model 3: bifactor	150.668	42	3.587	0.975	0.961	0.059	0.025	–	–	–	–
**Measurement invariance**											
**Measurement invariance across genders**											
Model 4: bifactor for boys (*n* = 365)	79.674	42	1.897	0.984	0.974	0.050	0.025	–	–	–	–
Model 5: bifactor for girls (*n* = 389)	103.811	42	2.472	0.971	0.954	0.062	0.030	–	–	–	–
Model 6: configural invariance	183.485	84	2.184	0.978	0.965	0.056	0.028	–	–	–	–
Model 7: metric invariance	221.13	108	2.048	0.974	0.969	0.053	0.044	8VS7	37.645 (24)	1.569	−0.004
Model 8: scalar invariance	244.255	116	2.106	0.971	0.967	0.054	0.045	9VS8	23.125 (8)	2.891	−0.003
Model 9: error invariance	267.379	128	2.089	0.969	0.968	0.054	0.051	10VS9	23.124 (12)	1.927	−0.002
**Measurement invariance across ages**											
Model 10: bifactor for age 12–13 (*n* = 199)	62.151	42	1.480	0.978	0.966	0.049	0.036	–	–	–	–
Model 12: bifactor for age 14–15 (*n* = 213)	57.881	42	1.378	0.985	0.976	0.042	0.035				
Model 13: bifactor for age 16–18 (*n* = 338)	57.881	42	1.378	0.985	0.976	0.042	0.035	–	–	–	–
Model 14: configural invariance	207.992	126	1.651	0.977	0.963	0.051	0.034	–	–	–	–
Model 15: metric invariance	291.965	180	1.622	0.968	0.965	0.058	0.044	4VS5	83.185 (54)	1.540	−0.009
Model 16: scalar invariance	301.158	190	1.585	0.968	0.967	0.048	0.059	5VS6	7.268 (10)	0.727	−0.000
Model 17: error invariance	336.748	214	1.574	0.965	0.967	0.048	0.061	6VS7	35.672 (24)	1.486	−0.003

*RMSEA, root mean squared error of approximation; SRMR, standardized root mean square residual; CFI, Comparative fit index; TLI, Tucker-Lewis index.*

**TABLE 3:** Standardized factor loadings for the bifactor model of NPOQ.

Items	General: NPO	F1	F2	F3	I-EVC
1	0.43	0.49			0.44
8	0.60	0.46			0.63
12	0.63	0.48			0.63
2	0.64		0.36		0.76
3	0.70		0.20		0.92
5	0.76		0.49		0.71
9	0.72		0.23		0.91
4	0.66			−0.37	0.76
6	0.77			−0.26	0.90
7	0.70			0.11	0.98
10	0.62			0.32	0.79
11	0.72			0.44	0.73
*ω*	0.93	0.77	0.86	0.85	
*ω_*H*_*/*ω_*HS*_*	0.87	0.33	0.15	0.01	

*ω, omega; ω_*H*_, omegaH; ω_*HS*_, omegaHS; I-ECV, Item explained common variance.*

### Evaluation of the Bifactor Model

Seven indices including coefficient omega (*ω*), coefficient omega hierarchical (*ω_*H*_*), coefficient omega hierarchical subscale (*ω_*Hs*_*), explained common variance (ECV), item for ECV (I-ECV), and percent uncontaminated correlations (PUC) were used to further evaluate the bifactor model (Rodriguez et al., 2016a,b). The coefficient omega (*ω*) estimates the proportion of variance in the total score attributable to all “modeled” sources of common variance. The *ω* for the general factor was 0.93, and the three domain-specific factors were 0.77, 0.86, and 0.85. Thus, both the total scale and three domain-specific factors had good consistent reliability. The coefficient hierarchical omega (*ω_*H*_*) reflects the proportion of variance in the NPOQ scores attributable to the general factor. The ω*_*H*_* for the general factor was 0.87, which was larger than the criterion (*ω_*H*_* > 0.50; Reise, 2012; Gu et al., 2017), indicating that 87% of the variance in the composite NPOQ scores was attributable to the general factor and the homogeneity of the NPOQ was high. Thus, synthesizing the total scores for the NPOQ was meaningful. The coefficient omega hierarchical subscale (ω*_*HS*_*) reflects that the proportion of variance in the scores was attributable to each domain-specific factor, independent of the general factor. The ω*_*HS*_* values were as follows: Factor 1 (perceived threat) = 0.33, Factor 2 (self-inefficacy) = 0.15, and Factor 3 (negative outcome expectancy) = 0.01. Further calculating the ratio of ω_*HS*_ divided by ω showed that the variation contributed by group factors were 42.9, 17.4, and 1.2%. These results indicated that the homogeneity of Factor 3 (negative outcome expectancy) was relatively low.

ECV quantifies the amount of common variance attributable to general and domain-specific factors. In this study, the general factor accounted for 78% and domain-specific factor explained 22% of the common variance, indicating that the common variance was largely spread across the general NPO. Similarity, The average I-ECV value was 0.76 (values ranged from 0.44 to 0.98, see Table 2), with only four items (items 3, 6, 7, and 9) evidencing I-ECV values larger than 0.80. PUC characterizes the percentage of NPOQ item correlations contaminated by variance attributable to the general and domain-specific factors. The PUC value for the NPOQ was 0.71. The high I-ECV and PUC indicate that many item correlations were attributable to the general factor.

### Measurement Invariance of the NPOQ

The results of the measurement invariance test and the fits for all models are summarized in Table 2. We initially separately examined the fits of the bifactor model to the sub-groups (Schmitt and Kuljanin, 2008; Chen et al., 2012). And the bifactor model showed an adequate model fit for boys and girls (see Models 4 and 5), which was a prerequisite for testing the measure of invariance across gender. The model testing for configural invariance (Model 6) achieved an acceptable fit, indicating that both girls and boys possessed the same bifactor structure for the NPOQ. Following this, all factor loadings were constrained to be equal across genders (Model 7). Comparing Model 7 with Model 6 showed that *χ^2^* significantly increased (*p* = 0.038) but *χ^2^*/*df* was less than 5 (1.569); *CFI* decreased less than 0.01 (0.004), which supported metric invariance across girls and boys. Model 8 added constraints to intercepts, resulting in a significant *χ^2^* of 23.125 for 8 *df* (*p* = 0.003). However, *χ^2^*/*df* was smaller than 5 (2.891) and *CFI* decreased less than 0.01 (0.003), supporting scalar invariance across girls and boys. Model 9 constrained the error variances to be equivalent across gender, resulting in a significant *χ^2^* of 23.124 for 12 *df* (*p* = 0.027) but small *χ^2^*/*df* (1.927 < 5) and *CFI* (0.002 < 0.01), supporting error invariance across girls and boys. Thus, a strict measurement invariance across genders was supported.

Measurement invariance across age was also tested. As showed in Table 2. The bifactor model showed well model fit for age at 12–13, 14–15, and 16–18 (see Models 10, 12, and 13), which was a prerequisite for testing the measurement invariance across age. The model testing for configural invariance (Model 14) achieved an acceptable fit, indicating that three age groups possessed the same bifactor structure for the NPOQ. Following this, all factor loadings were constrained to be equal across age (Model 15). Comparing Model 14 with Model 15 showed that *χ^2^* significantly increased (*p* = 0.007) but *χ^2^*/*df* was less than 5 (1.540); *CFI* decreased less than 0.01 (0.009), which supported metric invariance across age. Model 16 added constraints to intercepts, resulting in a significant *χ^2^* of 7.268 for 10 *df* (*p* = 0.700). And *χ^2^*/*df* was smaller than 5 (0.727) and *CFI* decreased less than 0.01 (0.000), supporting scalar invariance across age. Model 17 constrained the error variances to be equivalent across age, resulting in a significant *χ^2^* of 35.672 for 24 *df* (*p* = 0.059) and small *χ^2^*/*df* (1.486 < 5) and *CFI* (0.003 < 0.01), supporting error invariance across age. Thus, a strict measurement invariance across age was supported.

### Structural Regression Model

The incremental validity of the NPOQ’s domain-specific factors over the general factor was examined using a structural regression model that simultaneously modeled the bifactor model of the NPOQ, bifactor model of the PSWQ, and three-factor model of the DASS. The structural regression model provided an adequate fit to the data (*χ^2^* = 2621.425, *df* = 1065, *χ^2^*/*df* = 2.461; *RMSEA* = 0.046, *CFI* = 0.915; *TLI* = 0.906; *SRMR* = 0.056). The standardized beta weights from the structural regression model are presented in Table 4. The general factor NPO accounted for the main variance in engagement with worry, depression, anxiety, and stress. Moreover, Factor 1 (perceived threat) was positively correlated with engagement with worry and stress, while Factor 2 (self-inefficiency) and Factor 3 (negative outcome expectancy) accounted for unique variances in the general factor of the PSWQ.

**TABLE 4:** Standardized beta weights from structural regression model.

NPOQ factor	PSWQ-factor (criterion variable)			DASS-factor (criterion variable)		
		
	Engagement of worry	Absence of worry	G: Worry	Depression	Anxiety	Stress
F1: perceived threat	0.12*	−0.05	0.05	0.05	0.11	0.12*
F2: self-inefficiency	0.02	0.10	0.31***	−0.08	−0.02	−0.06
F3: negative outcome expectancy	0.05	0.11	0.34***	−0.12	0.00	0.01
G: NPO	0.57***	0.06	0.11	0.62***	0.57***	0.62***

NPOQ, Negative Problem Orientation Questionnaire; DASS, The Depression Anxiety Stress scale; PSWQ, The Penn State Worry Questionnaire. *p < 0.05, ***p < 0.001.

## Discussion

The present study supported a bifactor model of the NPOQ in Chinese adolescents, which consists of one general and three domain-specific NPO factors including perceived threat, self-inefficacy, and negative outcome expectancy. Then multiple-group SEM showed that the bifactor model demonstrated strict invariance across gender and age. The results also showed that the general factor NPO accounted for the main variance in engagement with worry, depression, anxiety, and stress. Perceived threat was positively correlated with engagement with worry and stress, and both self-inefficiency and negative outcome expectancy accounted for unique variances in the general factor of the PSWQ. These results indicate that the NPOQ shows good construct and incremental validity for Chinese adolescents.

This study supported a bifactor model of the NPOQ for Chinese adolescents, which is better for our understanding the NPO. In the bifactor model of NPOQ, the general factor NPO captures the whole disruptive cognitive-emotional set for social problems, and the group factors describe special cognitive and emotional characters related to NPO. Of those, perceived threat involves a negative belief in social problem being a threat to health and wellbeing; self-inefficacy is a comprehensive cognitive-emotional set that includes self-doubt over one’s problem-solving ability and related negative emotional reactions such as low self-efficacy; and negative outcome expectancy mainly includes a negative emotional reaction of being pessimistic about outcome, and a cognitive threshold that is a low tolerance for frustration (Ladouceur et al., 1998; Robichaud and Dugas, 2005a). The bifactor model solves the contradiction between the unitary factor model and the concept of NPO in Western samples, and thus more fully reflects NPO’s connotations. From the bifactor model, NPO is not only defined as an entirely disruptive cognitive-emotional set or attitude toward social problems (i.e., a general factor), it is also described via three aspects: perceived threat, self-inefficacy, and negative outcome expectancy (i.e., group factors).

The bifactor model is also significantly useful when using the NPOQ in practice. First, it should consider the general NPO factor, for that accounts for the overwhelming amount of variance in the NPOQ. For this study, the substantial amount of reliable and common variances in the NPOQ was attributable to the general factor NPO (*ω_*H*_* = 0.85, *ECV* = 0.78); loadings on the general factor were nearly larger relative to the loadings on the group factors (see Table 4). The results indicated that the general NPO factor is a reliable variable and the NPOQ can be represented as a unidimensional model with little parameter bias. Second, the group factors should be considered, for they show special significance in the factor construct of the NPOQ. Both the perceived threat and self-inefficacy factors explained a certain proportion of reliable variance in the NPOQ (42.9 and 17.4%, respectively), and the two factors uniquely predicted engagement with worry, stress, or general worry (see Table 3). Thus, they can be considered separate from the general factor, and using the two group factors is warranted. Though it significantly predicted the worry symptoms, the negative outcome expectancy factor should not be independently interpreted from the general NPO factor, for its omega HS value was low (0.01).

This study also tested the measurement invariance of bifactor model of NPOQ. This study supports configural, metric, scalar, and error measurement invariance across gender and age. The results further evidence the construct validity of NPOQ, and suggest that the NPOQ is most accurately operationalized as consisting of a general factor and three domain-specific factors among both boys and girls, different age groups. Additionally, higher or lower scores on the NPOQ (both general and domain-specific factors) can be considered to reflect true group differences in the construct rather than the NPOQ having items that preclude sub-groups from responding in comparable ways (Vandenberg and Lance, 2000; Schmitt and Kuljanin, 2008). The results of this study provide the enough evidence to show the different NPO levels in boys and girls, different age group reflect the true group difference, which suggested that the NPOQ has the same construct across gender and age.

The incremental validity of the NPOQ was also tested with a structural regression model. The results mainly support the general factor NPO being positively correlated with depression, anxiety, and stress. This finding is similar to those of previous clinical studies that supported the important role of NPO in the development of anxiety and depressive disorders (Fergus et al., 2015; Koerner et al., 2015; Hearn et al., 2017). Moreover, this study also showed that the domain-specific factor of perceived threat was positively related to engagement with worry, and both self-inefficiency and negative outcome expectancy were positively associated with the general factor of worry. These results support the role of domain-specific factors of NPO in adolescent worry. They can be explained via the cognitive model of worry (Dugas et al., 2005; Dugas and Robichaud, 2007), which holds that negative beliefs regarding problems have salient effects on developing and maintaining worry. Specifically, the cognitive model of worry supposes that people with higher levels of NPO tend to consider problems as threats to their wellbeing and believe that social problems will result in negative outcomes; thus, they are usually less effective at problem solving (Robichaud and Dugas, 2005b). These negative cognitive and emotional sets lead people to avoid real problems and engage in worry (Dugas et al., 2005).

Several limitations in the study should be acknowledged. Firstly, this study only used few and limited external criterion variables. It is possible that the domain-specific factors in NPOQ may uniquely contribute to criterion variables not assessed in the study. Secondly, the criterion variables in the study were self-report measures that were fulfilled concurrently with NPOQ. Moreover, only the non-clinical adolescents sample was considered in this study. Future studies should explore it in clinical adolescent samples and survey in a longitudinal time to precisely test the predictive effects of general factor and domain-specific factors in NPOQ. Although existed outstanding limitations, this study provide support for a bifactor model of NPOQ with a general NPO factor and three domain factors in Chinese adolescents and measurement invariance across gender. The bifactor model not only provides adequate construct for NPOQ but also benefit to our understanding the concept of NPO in Chinese adolescents. This research also suggests that when using the NPOQ, the total score should be first considered, and its two sub-scales, perceived threat and self-inefficiency, should be analyzed subsequently because they have unique effects on the measure.

## Data Availability Statement

All datasets generated for this study are included in the article/supplementary material, further inquiries can be directed to the corresponding authors.

## Ethics Statement

The studies involving human participants were reviewed and approved by the Academic Committee of Fujian Normal University. Written informed consent to participate in this study was provided by the participants’ legal guardian/next of kin.

## Author Contributions

RL and HX conceived the original idea for the study and wrote the study manuscript. QW and SS managed the day to carry out the data collection. RL and YY supervised this study. All the authors participated in the conception and design of the work, read and approved the final manuscript, and believe that the manuscript represents valid work.

## Conflict of Interest

The authors declare that the research was conducted in the absence of any commercial or financial relationships that could be construed as a potential conflict of interest.
